# Machine‐Learning Models Trained on Genomic Summary Statistics Assess Extinction Risk and Recovery Potential of Bottlenecked Populations

**DOI:** 10.1111/eva.70331

**Published:** 2026-10-07

**Authors:** Johanna C. Winder, Jack N. Marsters, Thomas Birley, Jasmyn Gooding, Taoyang Wu, Jim J. Groombridge, Hernán E. Morales, Cock van Oosterhout

**Affiliations:** ^1^ School of Environmental Sciences University of East Anglia, Norwich Research Park Norwich UK; ^2^ Durrell Institute of Conservation and Ecology (DICE), School of Natural Sciences University of Kent Canterbury UK; ^3^ Mansfield College University of Oxford Oxford UK; ^4^ School of Computing Sciences University of East Anglia, Norwich Research Park Norwich UK; ^5^ Globe Institute University of Copenhagen Copenhagen Denmark

**Keywords:** biodiversity conservation, extinction, genomic data, machine learning, population recovery, SLiM simulations

## Abstract

Forecasting extinction risk from genomic data is challenging because of interactions among environment, demography, and genomic erosion. Here, we use known outcomes from forward simulations as a proof‐of‐concept study to test whether machine‐learning (ML) models can discriminate relative extinction risk and recovery potential from genomic summary statistics, and identify the most informative predictors. Using individual‐based, forward‐in‐time simulations, we generated genomic and fitness data for 11,200 populations across 112 demographic parameter combinations (ancestral population size: 100–10,000, bottleneck size: 4–50, fecundity: 8–64). We evaluated populations at three time points relative to bottleneck onset: 100 years before, 10 years before and at onset. We then assessed their subsequent fate up to 200 years after the end of the bottleneck, classifying populations as extinct or recovered. From 10 sampled individuals per population, we derived 738 predictors spanning diversity, heterozygosity, genetic/realised/masked load, and ROH‐based inbreeding (FROH). Unsupervised analyses primarily captured historical demography and did not separate outcomes. In contrast, supervised models achieved high performance at bottleneck onset, correctly predicting population fate (i.e., future extinction or recovery) from genomics alone with ≈86%–87% accuracy. However, extinction was harder to predict correctly than recovery because simulated extinctions were rarer. Bottleneck size and fecundity provided strong baseline predictability, with genomics adding modest but complementary information. The upper‐quantile ROH/FROH summaries were consistently more informative than mean diversity or mean load. A complementary single‐genome analysis showed that reference‐genome‐style summaries retain useful signal when demographic information is sparse. Together, these results highlight the promise of ML‐informed extinction‐risk assessment to translate genomic indicators into more actionable, early‐warning signals for biodiversity conservation.

## 1 Introduction

Biodiversity is declining at a rate that exceeds historical baselines, driven by interacting anthropogenic pressures such as habitat loss and fragmentation, overexploitation, pollution, emerging diseases and climate change (Tilman et al. 1994; Barnosky et al. 2011; Haddad et al. 2015; Trisos et al. 2020). These stressors reduce population sizes and increase extinction risk through increased demographic and environmental stochasticity. In addition, genetic processes can exacerbate this decline, and their impacts act most strongly in small, isolated populations (Frankham 2005; van Oosterhout, Helsen, et al. 2026; van Oosterhout, Speak, et al. 2026). Inbreeding, drift and reduced efficacy of selection can erode genetic diversity, increase homozygosity and elevate the realised load of deleterious variation (Charlesworth 2009; Luikart et al. 2010; Bertorelle et al. 2022; Dussex et al. 2023; van Oosterhout, Helsen, et al. 2026; van Oosterhout, Speak, et al. 2026). Empirical genomic studies increasingly report that demographic recovery does not necessarily imply genetic recovery, and that ‘hidden’ genetic risks may persist after conservation interventions (e.g., Jackson et al. 2022; Femerling et al. 2023; Fontsere et al. 2025). This is consistent with classical population‐genetic theory: demographic recovery does not by itself restore lost variation or remove fixed deleterious alleles. This disconnect between census numbers and genomic condition is a real challenge in conservation genetics, motivating approaches that can integrate many partially informative genomic indicators, especially when demographic and ecological data are incomplete (van Oosterhout 2024). To tackle this challenge, we need to identify which *combination* of genomic indicators provides quantitative information about future population viability and extinction risk.

Recent work on genomic erosion illustrates both the promise and the challenge of translating genomic data into conservation‐relevant information (Wilder et al. 2023). Using single reference genomes from 240 placental mammals in the Zoonomia dataset, Wilder et al. (2023) showed that historical effective population size is associated with contemporary heterozygosity and with proxies for deleterious genetic load, and that machine learning (ML) models based on these genomic summaries can predict broad IUCN threat status. This is particularly useful for data‐deficient taxa where census and ecological information are sparse. However, the study also showed that the predictive value of genomics is modest when detailed ecological information is already available. Specifically, the ML models by Wilder et al. (2023) that incorporated genomic window–based features did not outperform models built from ecological predictors. This result underscores the central methodological challenge for genomics‐informed risk prediction: rather than asking whether genomic data correlate with IUCN Red List threat status, we need to identify when genomic indicators add independent information to enhance predictions of extinction risk beyond standard demographic and ecological covariates (van Oosterhout 2024).

Recent conservation genomics studies of populations that narrowly avoided extinction have revealed signatures of long‐term drift, elevated runs of homozygosity (ROH) and shifts in deleterious variation that may constrain adaptive potential and elevate future risk (e.g., Feng et al. 2019; Jackson et al. 2022; Femerling et al. 2023; Fontsere et al. 2025; Grossen et al. 2020; Hoffman et al. 2024; Kleinman‐Ruiz et al. 2022; Mathur and DeWoody 2021; Calderón et al. 2024; Kyriazis et al. 2026). Likewise, habitat loss and fragmentation can create systematic trajectories of genomic erosion by reducing gene flow and effective population size, linking landscape change to predictable genomic consequences (Pinto et al. 2024). However, comparative studies show that genomic erosion is heterogeneous among taxa, suggesting that differences in demographic history, recombination landscapes, and selection regimes can only be captured reliably by assessing multiple metrics (Wang et al. 2026). In parallel, conceptual syntheses emphasise that the dynamics of genetic load, realised load and masked load under population decline and recovery can be counterintuitive: purging, drift and the exposure of recessive deleterious alleles challenge the simple paradigm that ‘more homozygosity equals higher risk’ (Dussex et al. 2023). Together, these findings motivate two methodological needs: (i) predictive frameworks that can integrate multiple genomic and demographic indicators, and (ii) rigorous evaluation of which modelling approaches best capture extinction‐relevant signals in these complex data.

Machine learning (ML) provides a natural toolkit for this integration problem. Supervised ML methods can combine large numbers of predictors, represent nonlinear relationships and interactions, and generate probabilistic classifications that may be more useful for decision‐making than single‐threshold rules (Olden et al. 2008). ML has therefore been increasingly adopted in ecology for prediction and classification tasks, including species distribution modelling, trait inference and conservation prioritisation (Branco et al. 2023; Tuia et al. 2022; Silvestro et al. 2022). However, genomic prediction of extinction risk faces four challenges. First, ground‐truth labels for extinction are rare and noisy in real systems: ‘extinct’ outcomes are uncommon, occur over long‐time horizons, and are confounded by varying conservation management actions and observation bias (i.e., extinct species tend to be underrepresented in conservation genomic studies). Second, genomic predictors are heterogeneous in scale and meaning, ranging from diversity summaries to ROH distributions and genetic load proxies. Empirical estimates of inbreeding depression are often more directly linked to demographic performance than genomic summary statistics, but such fitness data are unavailable for many taxa. Third, their relationships to fitness and persistence are often indirect and time‐lagged (Dussex et al. 2023; Pinto et al. 2024). Fourth, evaluation must confront class imbalance and the high costs of consequential errors. In conservation, false negatives (predicting persistence when extinction is likely) can be particularly consequential. Performance metrics that emphasise the extinct class (e.g., precision‐recall summaries) are therefore often more appropriate than overall accuracy or receiver operating characteristic (ROC)‐based discrimination alone (He and Garcia 2009; Saito and Rehmsmeier 2015).

Forward‐in‐time population genomic simulation offers a practical route to address these challenges and to benchmark predictive pipelines under controlled conditions. Simulation platforms such as SLiM allow explicit modelling of demography, recombination and selection, generating synthetic populations in which the mechanisms are known, and the outcomes can be observed directly (Haller and Messer 2023). Critically, simulations can produce binary outcomes (recovery vs. extinction) under replicated runs. Using these distinct classes enables supervised learning with true labels and permits principled comparisons among modelling choices: feature sets, aggregation strategies, algorithms, and the timing of prediction relative to demographic change. Simulations therefore provide a framework to test which genomic summaries are most informative. Furthermore, it allows us to assess how robust they are across parameter regimes, and to compare different ML approaches. Any inference from this framework is conditional on the assumed genetic architecture, recombination model, life history and route by which fitness affects demography in our computer simulations. In other words, this study benchmarks one biologically motivated conceptual scenario rather than providing a case study for a specific species or proposing a universal model of extinction.

Here we develop and benchmark a ML pipeline for predicting extinction outcomes from genomic and demographic information using replicated forward simulations spanning a broad set of bottleneck scenarios. We treat these known simulated outcomes as a controlled benchmarking framework for testing whether genomic summary statistics contain usable signal about relative extinction risk and recovery potential. Building on the emerging empirical picture of genomic erosion during decline and after recovery (Jackson et al. 2022; Femerling et al. 2023; Fontsere et al. 2025) and the conceptual understanding of load dynamics under changing population size (Dussex et al. 2023), we use simulated genomes to ask when and how accurately extinction risk can be predicted. The parameter grid was designed to include severe near‐collapse bottlenecks observed in bird populations that have approached extinction, including species such as the Mauritius kestrel (minimum of four individuals; Groombridge et al. 2001), Seychelles paradise flycatcher (28 individuals; Femerling et al. 2023), pink pigeon (around 10 individuals; Jackson et al. 2022), and whooping crane (around 20 individuals; Fontsere et al. 2025). These examples illustrate the severity of the bottlenecks represented in our simulation framework. Several of these populations persisted only because of intensive conservation intervention. We focus on three aims. First, we compare a range of supervised algorithms that are widely used in ecological prediction, explicitly evaluating performance under imbalance with metrics that reflect extinct‐class detection. Second, we briefly explore feature engineering strategies, particularly distributional summaries across windows (e.g., quantiles of ROH and related statistics). Third, we assess how genomic predictors perform both with and without demographic covariates, and how predictive performance changes across timepoints before the bottleneck. The latter is directly relevant to early‐warning applications that might identify risk of population collapse before it occurs. By providing a transparent, reproducible benchmarking framework, our goal is to assess the potential of ML‐based, genomics‐informed extinction risk assessment in conservation.

## 2 Methods

### 2.1 Forward‐Time Simulations

We generated synthetic population genomic datasets using forward‐time simulations in SLiM 4 (Haller and Messer 2023). We simulated 112 parameter combinations spanning (i) the ancestral carrying capacity, that is, the census population size before population decline (*N*_a_ = 100, 200, 500, 1000, 2000, 5000, 10,000), (ii) carrying capacity during the bottleneck, which we refer to as the bottleneck size (*N*_b_ = 4, 10, 20, 50), and (iii) fecundity (offspring per pair per generation = 8, 16, 32, 64). These bottleneck sizes were chosen to span extreme near‐collapse scenarios relevant to some of the most severely bottlenecked bird populations. Note that the actual size of the population may be lower than *N*_b_ in cases where the population declined below its simulated carrying capacity.

For each parameter combination we ran 100 replicate populations (total = 11,200 populations). All populations had separate sexes and non‐overlapping generations. Mate pairs were monogamous and selected at random. Each mate pair produced a number of offspring equal to the simulation's fecundity parameter. Because generations were non‐overlapping and each pair reproduced once, this parameter represents offspring production per pair over its simulated reproductive lifetime. We explored fecundity values ranging between 8 and 64, comparable to previous studies (e.g., Pérez‐Pereira et al. 2022). Genotype‐derived fitness entered the non‐Wright‐Fisher life cycle through viability selection only, and selection was applied equally to both sexes. In SLiM, individual fitness was calculated multiplicatively across deleterious mutations, such that homozygous mutations contributed (1 + *s*) and heterozygous mutations contributed (1 + *hs*), where *s* and *h* are the selection and dominance coefficient, respectively (Figure S3). Each generation was implemented across two SLiM cycles. In the first cycle, random monogamous mating produced offspring. The parental generation was assigned zero fitness to enforce non‐overlapping generations. Offspring survival was then determined by their genotype‐derived fitness, following the default nonWF interpretation of fitness as survival probability in SLiM (Haller and Messer 2023). In the second cycle, reproduction was blocked and carrying capacity was enforced independently of genotype: if population size exceeded *K*, (*N − K*) individuals were chosen at random to die, whereas if *N* ≤ *K* all surviving individuals progressed to the next reproductive cycle. Thus, carrying capacity did not modify genotype‐based survival itself, but set the upper limit on how many individuals could persist to reproduce in the next generation. No additional selection acted on reproductive success or fecundity. For downstream feature generation, individuals were sampled in the late phase of the second SLiM cycle in the focal generation. If *N* > 10, we sampled 10 diploid individuals at random without replacement; if *N* ≤ 10, all extant individuals were sampled. In summary, this is a non‐Wright‐Fisher model with behaviour similar to that of a Wright‐Fisher model that allows for extinction to occur.

Each replicate followed the same demographic trajectory (in these simulations, generations are equal to years). Populations started to decline linearly at year‐100 (y‐100) for 100 years. At year 0 (y0) they entered a bottleneck with carrying capacity (*N*_b_) lasting 10 years (y10). They subsequently were allowed to recover linearly to a carrying capacity of *N*_r_ = 500. Population fate was binary; replicates that did not go extinct reached *N*_r_. These extinction/recovery labels therefore represent benchmark outcome classes within the simulated framework for supervised learning, rather than estimates of extinction probability in real populations. Population genetic metrics were collected 100 and 10 generations before the start of the bottleneck (y‐100 and y‐10, respectively), at the start of bottleneck (year 0; y0), as well as 10, 20, and 210 generations after the start of the bottleneck (y10, y20, y210).

### 2.2 Genome Model and Mutational Processes

The simulation used a hybrid genomic architecture with two distinct components. The first component was a linked 7.5 Mb sequence scaffold based on the length and coordinate structure of collared flycatcher chromosome 23 (Kawakami et al. 2017). This scaffold was used to represent contiguous sequence variation and to calculate ROH and FROH. It contained approximately 7.3% exonic, 39.9% intronic and 52.8% non‐coding sequence; deleterious mutations occurred in exonic and intronic regions, whereas the non‐coding fraction contained only neutral mutations. The second component was a reduced gene‐based functional component used to augment genetic load and contribute to variation in individual fitness. This component comprised five independently recombining blocks, each containing 1000 genes of 1500 bp. These blocks were not subdivisions of chromosome 23 and were not used for ROH detection or FROH calculation. The scaffold and functional blocks occupied one continuous SLiM coordinate system, but a recombination probability of 0.5 between the scaffold and the first block, and between successive blocks, made them effectively separate linkage units. Thus, the chromosome scaffold and the functional component served complementary purposes: (1) the linked scaffold provided the contiguous sequence used for ROH‐based inbreeding metrics, (2) the additional functional blocks increased the number of deleterious mutations contributing to variation in fitness. Together, the scaffold and additional functional blocks were parameterised to generate a genetic load representative of bottlenecked threatened species (Jackson et al. 2022). The per base mutation rate was set to *u* = 2 × 10^−8^. Note that this value is higher than pedigree‐based mutation‐rate estimates for collared flycatcher because the simulated genomic target was much smaller than a full avian genome, which kept the model computationally tractable. The higher rate was used to generate a plausible genetic load of between circa 2 and 8 lethal equivalents (Figure S2), which is a conservative range relative to meta‐analytic estimates of inbreeding depression (O'Grady et al. 2006). There was a two‐stage burn‐in; the first stage was for neutral mutations (run for 10*N*_a_ generations), the second burn‐in was for deleterious mutations (run for 10,000 generations). These conditions sufficed to establish mutation–selection–drift equilibrium.

Within the linked scaffold, mutations were assigned as neutral or deleterious in coding and intronic regions using a neutral to deleterious ratio of 3:2, and no deleterious mutations were permitted in the non‐coding fraction (Huber et al. 2017). The five additional functional blocks contained deleterious mutations only, introduced at the same deleterious mutation rate and with the same distribution of fitness effects as deleterious mutations in the scaffold exons. The linked 7.5‐Mb scaffold used a variable recombination map derived from collared flycatcher chromosome 23, with local recombination rates ranging from 6.54 × 10^−9^ to 3.55 × 10^−7^ per bp per generation in approximately 200‐kb intervals, giving a map length of approximately 100.6 cM. Within each functional block, recombination occurred at 2 × 10^−9^ per bp per generation within genes and with a probability of 0.002 at boundaries between neighbouring genes. A recombination probability of 0.5 between the scaffold and first functional block and between successive blocks made these effectively separate linkage units.

Selection coefficients (*s*) for deleterious mutations were drawn from a gamma distribution with mean *s* = −0.05 and shape parameter 0.5, following Kardos et al. (2021) and subsequent implementations by Dussex et al. (2023), Speak et al. (2024) and Al Hikmani et al. (2024). Five percent of deleterious mutations were assigned a lethal effect, *s* = −1. Dominance coefficients (*h*) were assigned as *h* = 0.5exp(−13|*s*|), so that more strongly deleterious mutations were increasingly recessive, consistent with the empirical relationship between dominance and fitness effects (Agrawal and Whitlock 2011). The distribution of fitness effects (DFE) was dominated by weakly to moderately deleterious mutations with a tail of stronger effects, including a fraction of lethal recessives. Dominance decreased with increasing deleterious effect size, such that more deleterious mutations were more recessive. We used this generalised genomic architecture to maintain comparability with our published and ongoing SLiM simulation work; simulation models necessarily rely on simplifying assumptions, and a more species‐specific mutation or recombination landscape was not required for the present proof‐of‐concept aims. The distribution represents a mutation–selection–drift equilibrium DFE, which is intentionally conservative, because it is likely to underrepresent the frequency of extreme‐effect deleterious mutations relative to some recent empirical inferences. For example, an elevated frequency of recessive lethal variants has been reported for the recovered 'Alalā population (Kyriazis et al. 2026). This conservative parameterisation is also consistent with our simulated genetic load of circa 2–8 lethal equivalents (Figure S2), which falls below the meta‐analytic estimate of 12.3 diploid lethal equivalents for inbreeding depression in wild populations (O'Grady et al. 2006). We consider this conservative approach appropriate here because it reduces the risk of overstating the contribution of genetic load in this proof‐of‐concept benchmarking study. Alternative DFEs, including species‐specific parameterisations, will be explored in future work as additional empirical data become available.

### 2.3 Individual‐Level Genomic Statistics

For each sampled individual, as well as at the population level, we computed genomic features in 15 non‐overlapping 500 kb segments across the 7.5 Mb chromosome. Within each segment we calculated:

- *Genetic load* as the sum of selection coefficients across deleterious mutations in the segment (Bertorelle et al. 2022).
- *Realised load* as the sum of selection coefficients for homozygous deleterious mutations plus *s* · *h* for heterozygous deleterious mutations (Bertorelle et al. 2022).
- *Masked load* as the complement of realised load (deleterious effects carried but not expressed as homozygotes) (Bertorelle et al. 2022).
- *Observed heterozygosity* (*H*_o_). For each individual, *H*_o_ was calculated as the proportion of sites within each 500‐kb segment at which its two chromosome copies differed.
- *Nucleotide diversity* (*π*), where applicable from segregating variation summaries. Population‐level nucleotide diversity (*π*) was calculated within each segment as the mean number of pairwise sequence differences per site among the 20 chromosome copies from the 10 sampled diploid individuals.
- ROH‐based inbreeding quantified as FROH, calculated as the fraction of the genome in ROH above specified length thresholds. This window‐based design was used for feature engineering, not because any single window was assumed to determine fitness in isolation. It allowed the machine‐learning models to test whether within‐genome heterogeneity in ROH, diversity, and deleterious variation across the scaffold improved prediction beyond simple mean summaries. This is analogous to decomposing a composite trait into component measurements: the aggregate value remains biologically meaningful, but the distribution of its components may contain additional predictive information. We also calculated FROH across the whole 7.5‐Mb scaffold (see below).

Genetic load was defined as the total burden of deleterious variation, realised load as the expressed burden in homozygotes and partially recessive heterozygotes, and masked load as the potential fitness loss of partially recessive deleterious alleles carried in heterozygotes (Bertorelle et al. 2022). Load components used in the machine‐learning analyses were calculated exclusively within 15 non‐overlapping 500‐kb windows spanning positions 0–7.5 Mb of the linked scaffold. This was done to place load on the same window‐based feature framework used for the other scaffold‐derived genomic summaries, allowing the models to capture spatial heterogeneity across the 15 genomic segments. Deleterious mutations in the five additional functional blocks contributed to individual fitness but lay outside these windows and were not included in these window‐level load predictors. Thus, the genetic, realised, and masked load predictors reported here refer to deleterious variation on the linked scaffold rather than whole‐genome load.

To capture the time scale of inbreeding, FROH was computed over the whole 7.5 Mb chromosome, as well as for 500 kb windows. Runs of homozygosity (ROH) were identified from the simulated diploid genomes directly within SLiM as contiguous homozygous segments across the chromosome. The independently recombining functional blocks were not used for ROH detection or FROH calculation; all ROH and FROH metrics were calculated only on the linked 7.5 Mb chromosome scaffold. For each individual and genomic region, homozygous runs were first detected, after which runs shorter than specified length thresholds were excluded; the remaining runs were summed and divided by the length of the region to obtain FROH. For each 500 kb window, we used ROH cutoffs of 20, 50, and 100 kb. At the whole‐chromosome level, we used 200 kb, 500 kb, and 1 Mb cutoffs. These thresholds were scaled to the 7.5 Mb simulated scaffold so that different ROH classes captured more recent versus less recent autozygosity within this reduced genomic representation. They are therefore not intended to be transferred directly to empirical whole‐genome studies. We interpret these ROH thresholds as internally consistent feature classes within the reduced simulation scaffold, rather than as direct estimates of the age of autozygous segments in an empirical whole‐genome analysis. In this reduced scaffold, short‐to‐intermediate ROH thresholds were the most informative because they were more prevalent and therefore captured more among‐replicate heterogeneity. This does not imply that very long ROH are unimportant in general. Rather, in larger genome representations, or with more advanced feature engineering, rare but consequential very long ROH may become more informative. The design in the present study enabled comparison of local ROH distribution (in windows) and overall inbreeding signal (whole‐chromosome scale) in our simulated data, which were later evaluated for predictive utility.

### 2.4 Population‐Level Aggregation and Feature Construction

To build population‐level predictors, we aggregated individual statistics across the 10 sampled individuals per replicate. Specifically, for each replicate and each feature, we computed empirical quantiles across the 10 sampled individuals using the quantile function in R (including minima and maxima). This summarised within‐population dispersion and tail behaviour, which is expected to be informative under stochastic extinction dynamics. For example, a high upper quantile for FROH indicates that some individuals within a replicate carried unusually high autozygosity, which is evidence of recent inbreeding, even if the replicate mean FROH was moderate.

Across 15 segments, eight segment‐level features were summarised using quantiles (0, 0.2, 0.4, 0.6, 0.8, 1), and likewise, the three whole‐chromosome ROH features were summarised. Altogether, this resulted in 738 predictors per population. We assembled these predictors for multiple time points (see above).

### 2.5 Unsupervised Learning

To assess whether extinct and recovered replicates occupy separable regions of multivariate feature space without outcome labels, we performed principal component analysis (PCA) using scikit‐learn (Pedregosa et al. 2011) and visualised projections for each time point. We then applied *k*‐means clustering (*k* = 5) to PCA scores to quantify whether dominant structure reflected extinction outcome versus demography. Using *k* = 5 avoided forcing the structure in either of the two obvious a priori groupings: the two eventual outcome classes (extinct vs. recovered) or the seven ancestral population‐size (*N*_a_) classes.

To compare the geometry of extinct and recovered clouds in PCA space, we computed convex hulls for each class and quantified overlap of hull polygons. Convex hulls and polygon operations were implemented using standard computational geometry libraries in Python (Shapely), providing a scalar measure of class separation (lower overlap implies greater separability) across time points.

### 2.6 Supervised Machine Learning

Supervised classifiers differ in how they separate outcome classes. Logistic regression and linear discriminant analysis (LDA) are linear methods that fit a simple boundary in predictor space. Decision trees learn rule‐based splits of the predictors; random forests and extreme gradient boosting (XGBoost) combine many trees into an ensemble to capture non‐linearities and interactions (forests average trees, whereas boosting builds trees sequentially to correct previous errors). *k*‐nearest neighbours (kNN) is a distance‐based method that predicts an outcome from the most similar observations. Support vector classifier (SVC) finds a maximum‐margin separating boundary (optionally non‐linear via kernel functions) (see Pedregosa et al. 2011; Chen and Guestrin 2016). We benchmarked seven supervised classifiers:

1. Logistic regression.
2. Linear discriminant analysis (LDA).
3. Decision tree.
4. Random forest.
5. Extreme gradient boosting (XGBoost).
6. *k*‐nearest neighbours (kNN).
7. Support vector classifier (SVC).

### 2.7 Data Splitting, Preprocessing, and Class Imbalance

For each time point, we held out 20% of populations as a test set using stratified shuffling to preserve class proportions (scikit‐learn StratifiedShuffleSplit). The remaining 80% was used for model selection and training under 5‐fold cross‐validation.

Because extinction was the minority class in several time points, we accounted for class imbalance using class weighting (Benkendorf et al. 2023; He and Garcia 2009). Where required, predictors were scaled with StandardScaler. Scaling was evaluated explicitly because distance‐based (kNN) and margin‐based (SVC) classifiers can be sensitive to feature scale, whereas tree‐based methods are typically robust.

### 2.8 Hyperparameter Tuning and Overfitting Control

For each classifier, we performed a grid search over algorithm‐specific hyperparameters (e.g., regularisation strength for logistic regression, tree depth and number of estimators for random forest, kernel and *C* for SVC, learning rate and boosting rounds for XGBoost) (Table S1). Hyperparameter tuning was performed within cross‐validation on the training set.

In addition to maximising predictive performance, we monitored overfitting using the difference between training and validation performance. Models exhibiting a > 5% train–test gap were treated as overfit and deprioritised in model selection. Final performance summaries were computed on the held‐out test set using the best hyperparameter configuration per algorithm and time point.

### 2.9 Performance Metrics

We summarised classifier performance using confusion matrices and threshold‐based metrics prioritised for rare‐event prediction. Specifically, we computed precision (positive predictive value for the extinct class), recall (sensitivity for the extinct class), and F1 score (harmonic mean of precision and recall) (Powers 2011). Where reported in the Results, we additionally summarised discrimination using threshold‐independent measures (ROC‐AUC and PR‐AUC), with PR‐AUC emphasised when extinction prevalence was low.

### 2.10 Demographic Baselines and Incremental Value of Genomics

To quantify how much predictive signal is available from demography alone, we fitted a baseline logistic regression using the demographic parameters available at bottleneck onset (notably *N*_b_ and fecundity) and evaluated performance under the same train/test splitting and cross‐validation scheme. Where included as a demographic predictor, population size refers to the realised census size (*N*) of the replicate at the focal sampling time, distinct from the specified carrying‐capacity parameters *N*_a_, *N*_b_ and *N*_r_. Because carrying capacity sets an upper limit rather than fixing population size, realised *N* could fall below the specified carrying capacity. We then trained the full set of genomic + demographic models at year 0 to assess whether genomic predictors improved performance relative to demography‐only baselines, and to identify cases where genomic information provided non‐redundant predictive power.

### 2.11 Feature Importance and Reduced‐Data Analyses

To interpret model predictions and identify which features were most influential, we quantified feature importance for the best‐performing non‐linear classifier using permutation‐based importance (Altmann et al. 2010). Permutation importance was computed on the held‐out test set by randomly permuting each predictor and measuring the resulting decrease in model performance, thereby ranking predictors by their marginal contribution to predictive accuracy under the fitted model.

To evaluate feasibility under constrained conservation‐genomics data scenarios (e.g., few individuals, partial predictors), we performed reduced‐data analyses in which models were trained on predefined subsets of features representing (i) genomic‐only predictors, (ii) demographic‐only predictors, and (iii) combinations omitting particular demographic variables (e.g., excluding *N*_a_ but retaining bottleneck and fecundity information). In addition, we examined whether key demographic parameters (*N*_b_, fecundity) could be approximated from genomic predictors by (a) fitting logistic regression models with demographic predictors alone and (b) computing correlations between demographic variables and genomic features to identify the strongest proxies.

To evaluate model performance when only a reference genome is available, we implemented a single‐genome analysis in which one diploid individual was sampled per simulated population replicate at each focal time point. This design avoids pseudo‐replication because each observation corresponds to a unique replicate and carries that replicate's extinction/recovery outcome label. For each sampled genome, we computed the same suite of genomic predictors used in the population‐level analyses (i.e., diversity, heterozygosity, genetic load components and ROH‐based inbreeding metrics derived from the simulated chromosome and windowing scheme), but without within‐population aggregation (no quantile summaries across individuals). We then trained and evaluated the same set of supervised ML classifiers and predictor subsets (‘test sets’, Table S1) as in the main analysis, allowing direct comparison of predictive performance between population‐sampled and single‐genome settings using identical features, preprocessing and evaluation metrics.

### 2.12 Software and Implementation Details

Simulations were run in SLiM 4 (Haller and Messer 2023). Data processing and modelling were implemented in Python (version 3.11) using scikit‐learn (Pedregosa et al. 2011) and XGBoost (Chen and Guestrin 2016), with supporting numerical routines from SciPy (Virtanen et al. 2020) where required. Population‐level quantiles were computed in R (4.3.1). Figures were produced using ggplot2 (R), seaborn and matplotlib (Python) and assembled for publication graphics.

## 3 Results

### 3.1 Simulation Output and Population Genetic Summary Statistics

Across 112 parameter combinations (ancestral population size *N*_a_ = 100–10,000; bottleneck population size *N*_b_ = 4–50; fecundity = 8–64), we generated 11,200 replicate populations (100 replicates per parameter set). For each population, 10 individuals were sampled and genomic variation was summarised at the onset of the bottleneck (year 0) into 738 features spanning nucleotide diversity (π), observed heterozygosity (*H*_o_), total, realised and masked load, and ROH‐based inbreeding coefficients (FROH) computed across 15 non‐overlapping 500 kb windows and at whole‐chromosome ROH length cut‐offs (Figure S1).

At year 0, several core genomic metrics primarily reflected ancestral population size. Nucleotide diversity and observed heterozygosity increased strongly with *N*_a_ (Spearman *R* ^2^ = 0.94 for both; Table 1, Figure 1a), whereas FROH showed moderate to strong negative associations with *N*_a_ across length cut‐offs (*R* ^2^ from −0.53 to −0.93; Table 1, Figure 1d). Load components showed distinct responses: total and masked load tended to increase with *N*_a_ (*R* ^2^ = 0.27 and 0.49, respectively), while realised load was negatively associated with *N*_a_ (*R* ^2^ = −0.43; Table 1, Figure 1b).

**TABLE 1:** Correlations between input parameters and groups of genomic features, associations between input features in extinct vs. recovered populations at the start of the bottleneck (year 0). Mean ± standard deviation of all input feature types was measured for the populations that (eventually) went extinct and those that recovered. Population means, averaged over all positions where relevant, were calculated. Spearman *R* ^2^ was used to identify correlations between these means and input features of ancestral population size (*N*_a_), bottleneck population size (*N*_b_) and fecundity. Difference in medians of features between populations that went extinct and recovered is shown. Mann–Whitney *U* test was significant (*p* < 0.05) for all differences due to large sample size. 95% confidence intervals were also included to report the magnitude and uncertainty of the difference between groups.

	Mean ± SD (extinct)	Mean ± SD (recovered)	*N*_a_ (*R* ^2^)	*N*_b_ (*R* ^2^)	Fecundity (*R* ^2^)	Difference in median (extinct v. recovered) 95% CI
Nucleotide diversity (*π*)	2.1 × 10^−4^ (±2.4 × 10^−4^)	1.6 × 10^−4^ (±2.1 × 10^−4^)	0.94	−0.01	−0.08	4.70 × 10^−3^ % [−7.30 × 10^−5^, 4.47 × 10^−3^]
Total genetic load	1.5 × 10^−1^ (±2.7 × 10^−1^)	1.4 × 10^−1^ (±2.6 × 10^−1^)	0.27	0.00	0.00	0.38% [0.37, 0.40]
Realised load	1.1 × 10^−2^ (±2.3 × 10^−2^)	1.2 × 10^−2^ (±2.4 × 10^−2^)	−0.43	0.01	0.07	−9.34 × 10^−3^ % [−1.1 × 10^−2^, −7.32 × 10^−3^]
Masked load	1.4 × 10^−1^ (±2.7 × 10^−1^)	1.2 × 10^−1^ (±2.6 × 10^−1^)	0.49	0.00	0.06	0.050% [0.043, 0.060]
FROH (20 kb)	5.1 × 10^−1^ (±3.8 × 10^−1^)	5.8 × 10^−1^ (±3.6 × 10^−1^)	−0.93	−0.01	0.09	−4.35% [−4.44, −4.26]
FROH (50 kb)	3.5 × 10^−1^ (±3.8 × 10^−1^)	4.0 × 10^−1^ (±3.7 × 10^−1^)	−0.87	0.01	0.09	−4.35 × 10^−3^ % [−5.74 × 10^−3^, −6.90 × 10^−3^]
FROH (100 kb)	2.6 × 10^−1^ (±3.6 × 10^−1^)	2.7 × 10^−1^ (±3.6 × 10^−1^)	−0.76	−0.02	0.09	−6.71 × 10^−4^ % [−3.56 × 10^−3^, −2.75 × 10^−3^]
FROH (200 kb)	2.5 × 10^−1^ (±3.0 × 10^−1^)	2.7 × 10^−1^ (±2.7 × 10^−1^)	−0.86	−0.03	0.11	−3.00% [−2.79, −3.25]
FROH (500 kb)	1.4 × 10^−1^ (±2.3 × 10^−1^)	1.2 × 10^−1^ (±1.8 × 10^−1^)	−0.72	−0.08	0.11	−4.79 × 10^−3^ % [−3.08 × 10^−3^, −5.31 × 10^−3^]
FROH (1 Mb)	7.7 × 10^−2^ (±1.6 × 10^−1^)	5.0 × 10^−2^ (±1.1 × 10^−1^)	−0.53	−0.12	0.10	1.59 × 10^−3^ % [6.82 × 10^−3^, 3.70 × 10^−3^]
Observed heterozygosity (*H*_o_)	2.1 × 10^−4^ (±2.4 × 10^−4^)	1.6 × 10^−4^ (±2.1 × 10^−4^)	0.94	−0.01	−0.08	3.25 × 10^−3^ % [1.21 × 10^−3^, 7.43 × 10^−3^]

**FIGURE 1:**
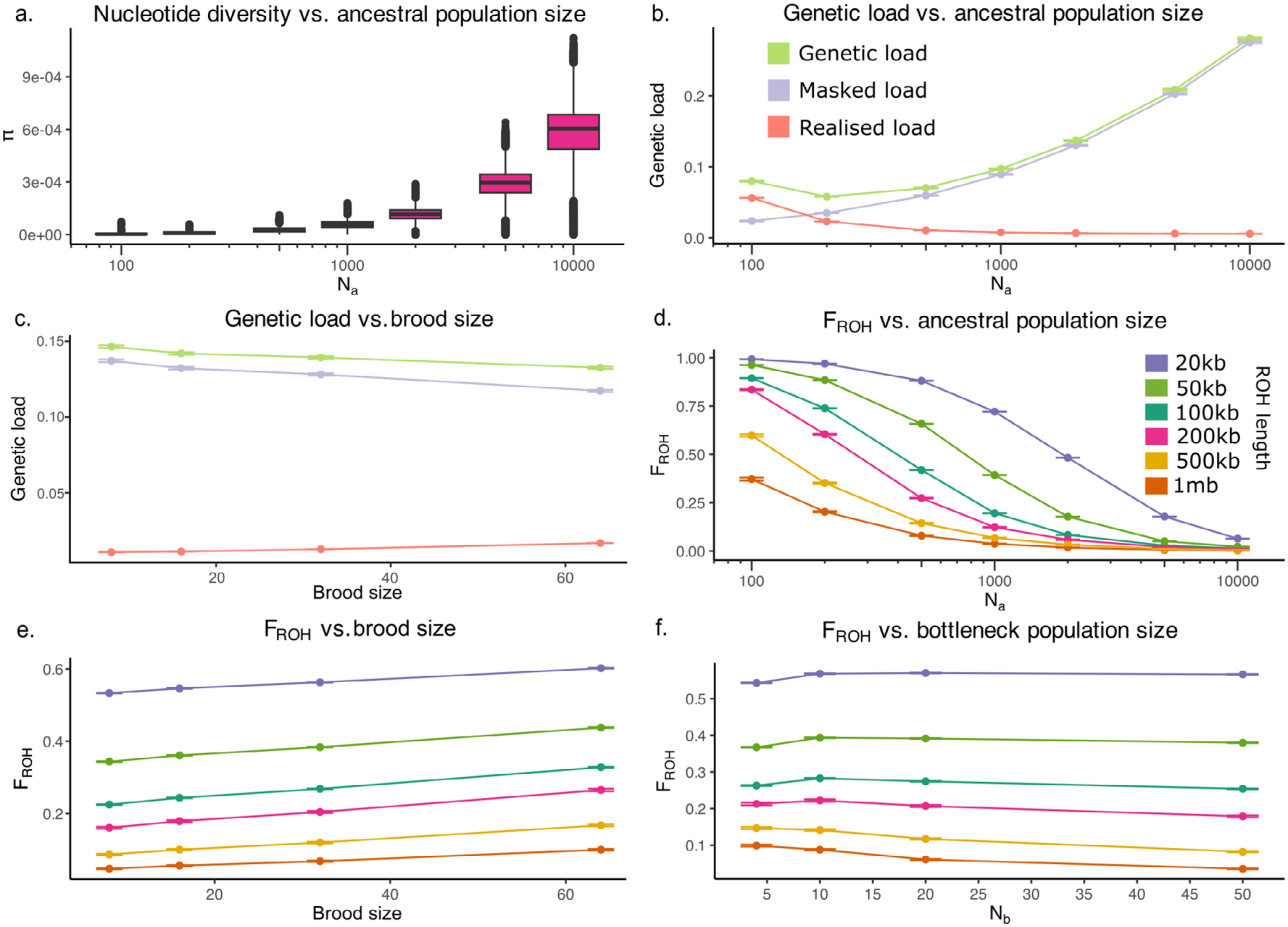
Effects of demographic parameter settings on core genomic metrics at bottleneck onset (y0) in SLiM simulations. Nucleotide diversity and load components were computed in 15 non‐overlapping 500 kb segments spanning the 7.5 Mb simulated chromosome; these segment‐based load values are therefore not directly comparable to genome‐wide totals. (a) Nucleotide diversity (*π*) plotted against ancestral population size (*N*_a_); boxplots show the median and interquartile range, with whiskers extending to 1.5 × IQR and points indicating outliers. Mean genetic load (green), realised load (red) and masked load (blue) across: (b) *N*_a_, and (c) fecundity. (d–f) Mean ROH‐based inbreeding, FROH (fraction of the genome in runs of homozygosity), across: (d) *N*_a_, (e) fecundity and (f) bottleneck size (*N*_b_), computed using multiple minimum ROH length thresholds (20 kb, 50 kb, 100 kb, 200 kb, 500 kb and 1 Mb; colours). For panels (b–f), lines connect means and error bars show 95% nonparametric bootstrap confidence intervals for the mean. Summaries are based on 11,200 replicate populations (112 parameter combinations × 100 replicates), marginalising over the remaining demographic parameters.

Genomic summaries at year 0 (y0) were only weakly associated with bottleneck size and fecundity: across feature groups, correlations with *N*_b_ and fecundity were small (|*R* ^2^| < 0.12 and < 0.11, respectively; Table 1, Figure 1). This weak association with fecundity is not unexpected, because fecundity was modelled here as an externally imposed demographic parameter rather than as a fitness‐dependent trait. It was still evaluated here because life‐history strategy has been proposed as an important correlate of long‐term effective population size and genome‐wide diversity (Chen et al. 2017; Romiguier et al. 2014). Differences between populations that ultimately went extinct versus recovered were generally modest at y0, with the largest reductions observed for FROH at shorter and intermediate ROH thresholds (e.g., FROH(20 kb) and FROH(200 kb); Table 1). Together, these patterns indicate that at bottleneck onset, most genomic summary statistics still primarily reflect ancestry and prior demographic history, whereas immediate viability is governed more directly by bottleneck size and fecundity. Furthermore, no single summary statistic provides strong separation of future outcomes at the start of the bottleneck, motivating multivariate prediction approaches evaluated below.

### 3.2 Unsupervised Learning Identifies Demography but Not Extinction Outcome

Principal component analysis (PCA) of the full feature set showed that populations did not form distinct clusters by eventual extinction outcome without prior labelling, irrespective of whether features were extracted 100 years before the bottleneck (y‐100), 10 years before the bottleneck (y‐10) or at the onset of the bottleneck (y0) (Figure 2a–c). Quantifying overlap between projected spaces using convex‐hull overlap indicated near‐complete overlap between extinct and recovered populations, with a marginally lower overlap at y0 (98.23%) than at y‐10 (99.23%).

**FIGURE 2:**
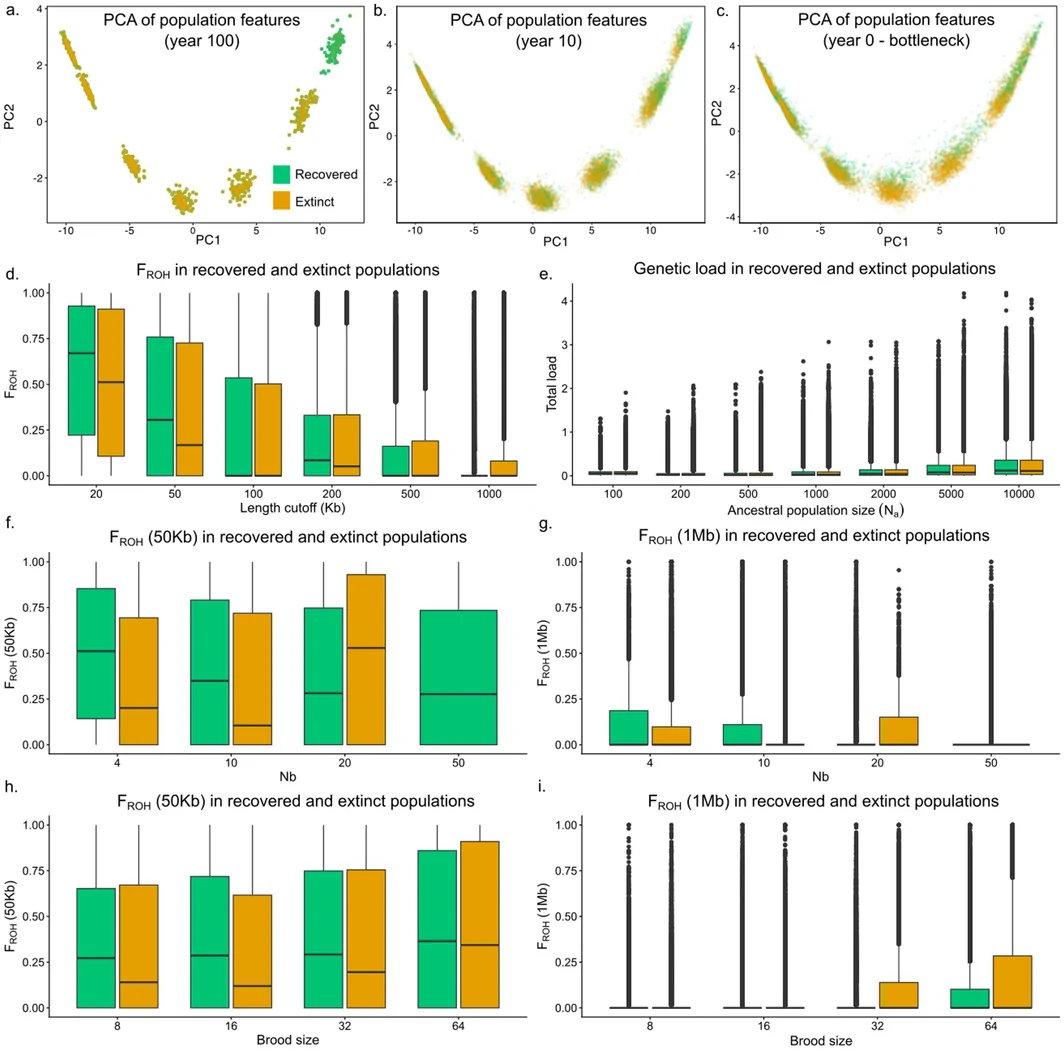
Genomic features of populations do not cluster by extinction risk and the distribution of key features differs only slightly between populations that go extinct and populations that recover. (a–c) Principal component analysis (PCA) of population‐level genomic feature vectors at (a) 100 years before the bottleneck (y‐100), (b) 10 years before the bottleneck (y‐10) and (c) at bottleneck onset (y0). Points represent replicate populations and are coloured by eventual outcome (recovered vs. extinct). The dominant PCA structure in panels a‐c reflects ancestral population size (*N*_a_); Figure 3 shows the same y0 PCA structure coloured by *N*_a_. (d) ROH‐based inbreeding, FROH (fraction of the genome in runs of homozygosity), summarised across minimum ROH length thresholds; boxplots show the median and interquartile range (IQR), with whiskers extending to 1.5 × IQR and points indicating outliers. (e) Total genetic load across ancestral population sizes (*N*_a_) for recovered and extinct populations (boxplot definition as in (d)). (f–i) FROH distributions across (f, g) bottleneck size (*N*_b_) and (h, i) fecundity. Panels (d–i) show features at the timepoint of bottleneck onset (y0).

Consistent with this, most individual feature groups differed little between extinct and recovered populations at y0 (typically < 0.5% difference in medians; Table 1). The remaining feature‐group summaries likewise showed extensive overlap between extinct and recovered populations, reinforcing that no single genomic feature class provided strong univariate separation of future outcomes at bottleneck onset (Figure 2e–i). Notable exceptions were the inbreeding coefficient based on ROH tracts (FROH 20 and 200 kb), which were lower in populations that later went extinct than in those that recovered by 4.35% (95% CI: 4.26–4.44) and 3.00% (95% CI: 2.79–3.25), respectively (Table 1, Figure 2d). Why do historically inbred populations tolerate bottlenecks better? The extinction rates across *N*_a_ provides important context for this counterintuitive pattern. At *N*_a_ = 100, 75.4% of replicates had already gone extinct before y0. Populations that survived until y0 were a strongly selected subset that could have purged the worst mutations. Among populations reaching y0, eventual extinction was 24.2%, 27.1%, 21.1%, 21.2%, 25.8%, 31.3% and 37.2% for *N*_a_ = 100, 200, 500, 1000, 2000, 5000 and 10,000, respectively. This pre‐bottleneck survivorship filtering, together with historical purging at low *N*_a_, likely left surviving populations with a lower masked load and greater tolerance of subsequent inbreeding, as observed in the Seychelles paradise flycatcher (Femerling et al. 2023).

In contrast, unsupervised clustering captured long‐term demographic signal. *K*‐means clustering in PCA‐reduced space recovered clusters that mapped closely to ancestral population size (*N*_a_) (Figure 3). Specifically, small ancestral populations (*N*_a_ = 100 and 200) were merged into a single cluster, very large ancestral populations (*N*_a_ = 5000 and 10,000) were merged into another, while intermediate values (*N*_a_ = 500, 1000 and 2000) formed distinct clusters.

**FIGURE 3:**
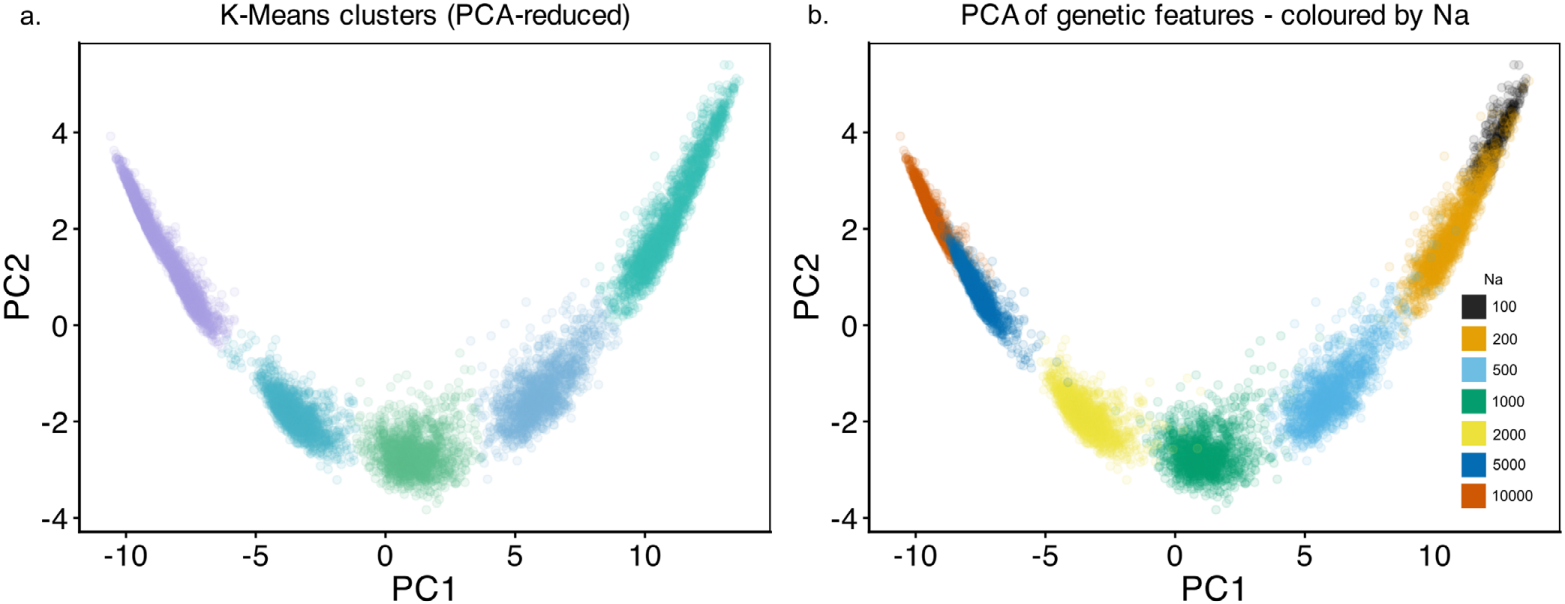
Unsupervised learning identifies ancestral population size (*N*_a_) from genomic features at the start of a bottleneck. (a) *K*‐means clustering with *k* = 5 partitions the simulated populations in principal component analysis (PCA)‐reduced genomic‐feature space, coloured by cluster. (b) PCA of genomic features at the start of the bottleneck (year 0) coloured by ancestral population size.

Together, these analyses indicate that, without supervision, genomic features predominantly encode historical demography (particularly *N*_a_) rather than the multivariate signal that distinguishes extinction versus recovery, motivating supervised learning approaches for extinction‐risk prediction.

### 3.3 Supervised Learning Can Predict Recovery, but Extinction Is Much Harder to Predict

We benchmarked seven supervised learning algorithms using genomic summary features at three timepoints relative to the bottleneck (y‐100, y‐10 and y0) to quantify how predictive information changes as the bottleneck approaches (Figure 4, Table 2). Overall performance depended strongly on timepoint. At 100 years pre‐bottleneck (y‐100), the best‐performing models (logistic regression and XGBoost) reached a 69.2% maximum accuracy of correctly predicting future population fate. Ultimately, 62% of populations recovered and 38% went extinct, so model accuracy exceeded the naive baseline (classifying all populations as recovered) by 7.2%.

**FIGURE 4:**
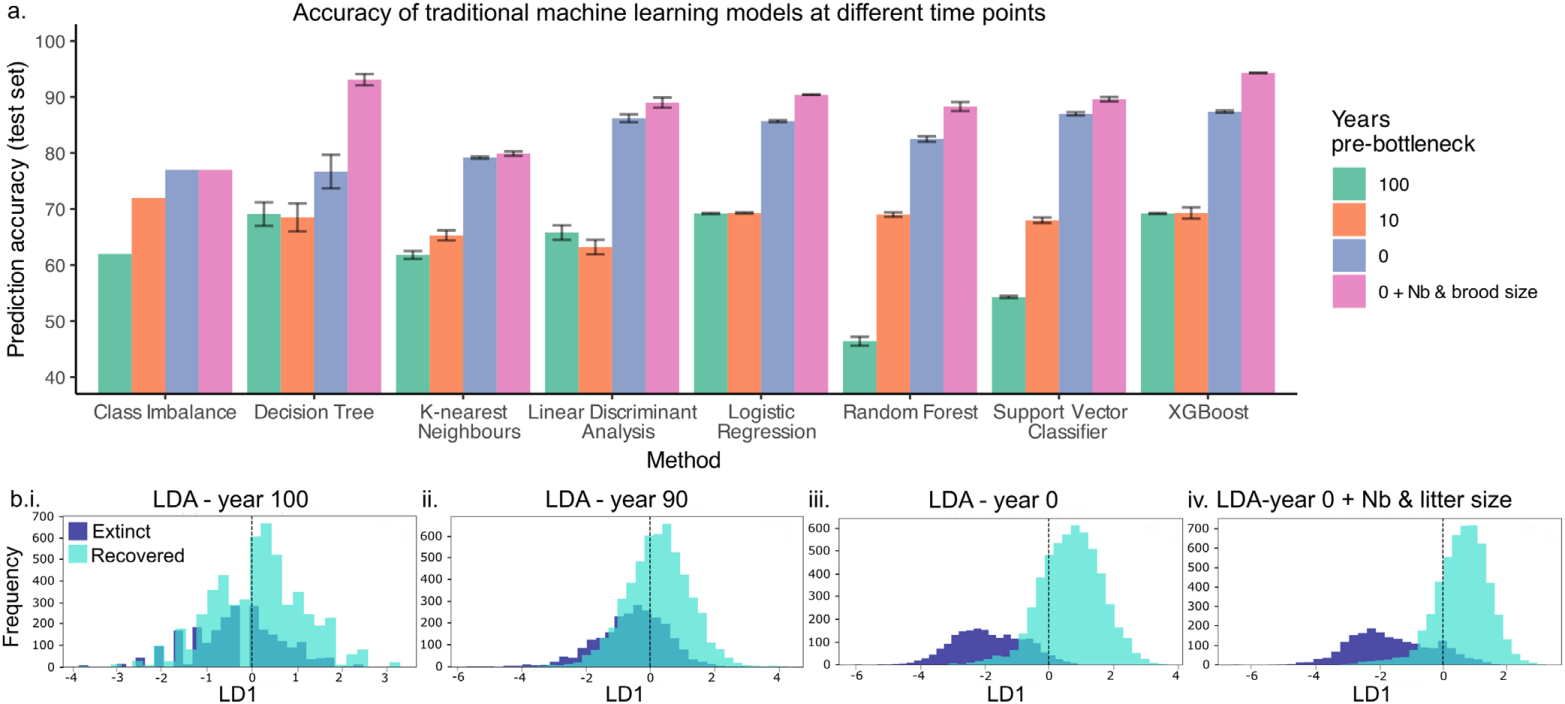
Performance of traditional supervised machine‐learning models to predict extinction risk from genomic features improves closer to a population bottleneck. (a) Accuracy of traditional machine‐learning models for predicting whether populations will go extinct based on genomic features. Models were trained on datasets from different timepoints prior to the start of a population bottleneck, and with the inclusion of bottleneck population size (*N*_b_) and fecundity information. Green—100 years before bottleneck, orange—10 years before bottleneck, purple—at the start of the bottleneck/year 0, pink—year 0 with the inclusion of *N*_b_ and fecundity. Error bars are training cross validation accuracy to account for variation in training dataset sampling. Most models show little to no improvement between years 100 and 10, but improve at the start of the bottleneck, and some models improve further with the inclusion of *N*_b_/fecundity. The class imbalance is included on the far left for reference of how random assignment would perform. Note the *y*‐axis scale does not start at 0%. (b) Linear class separation in 1 dimension with linear discriminant analysis improves in accuracy closer to the start of the bottleneck, with limited improvement with the inclusion of *N*_b_/fecundity. Extinct populations are in dark blue while recovered populations are in turquoise, with the decision boundary for classification marked with a dashed line.

**TABLE 2:** Precision, recall, F1 score and accuracy for best performing machine‐learning models to predict population extinction from genomic features, bottleneck population size and fecundity. Metrics are reported for performance on an unseen test dataset.

Inputs	Model type	Precision (E)	Recall (E)	F1 (E)	AUC ROC (R)	Overall accuracy
Genomic only	SVC	77.7	73.3	75.5	88.2	87.0
	XGBoost	87.1	63.3	73.3	88.2	87.4
	LDA	79.9	65.9	72.2	86.5	86.2
	Logistic regression	82.1	60.7	69.8	86.9	85.7
Genomic + *N*_b_ + fecundity	SVC	79.3	83.6	81.4	95.5	89.6
	XGBoost	89.0	90.4	89.7	98.2	94.3
	LDA	85.4	71.7	78.0	92.9	89.0
	Logistic regression	83.9	80.2	82.0	96.2	90.4
*N*_b_ + fecundity only	Logistic regression	63.1	92.9	75.2	84.0	83.3

At 10 years pre‐bottleneck (y‐10), recovery was more common (73% recovered) (note that 10.0% of populations went extinct during population size decline, pre‐bottleneck). By y0, 14.9% (1668/11,200) of replicates had failed to reach the bottleneck. Pre‐y0 extinction was strongly concentrated at *N*_a_ = 100 (75.4%), compared with 8.8%, 4.1%, 6.1%, 3.0%, 3.0% and 3.8% for *N*_a_ = 200, 500, 1000, 2000, 5000 and 10,000, respectively. Despite applying class weighting, no algorithm exceeded the baseline expected under the observed class imbalance, indicating limited discriminative signal in the genomic summaries at this pre‐bottleneck stage (Figure 4a).

In contrast, prediction accuracy increased substantially at the start of the bottleneck (y0). XGBoost achieved the highest overall accuracy (87.4% ± 0.30%; cross‐validation deviance), with linear discriminant analysis (LDA) and a support vector classifier (SVC) performing similarly (86.2% ± 1.1% and 87.0% ± 0.20%, respectively) (Figure 4a).

Because extinction was the less common outcome after y0, overall accuracy can be dominated by correct classification of the recovered (majority) class and therefore mask poor detection of extinction. Although the two class probabilities are complementary in a binary classifier, class‐specific precision, recall and F1 are not symmetric under class imbalance because they depend on the focal positive class and class prevalence. We therefore evaluated the best‐performing models using class‐sensitive metrics on an independent test set (precision, recall and F1 for each class, plus AUC/ROC; Figure 4b, Table 2). This revealed that the high accuracy of XGBoost was driven primarily by performance on the recovered class: its recall for extinct populations was comparatively low (63.3%). This indicates that populations that eventually went extinct were frequently misclassified as recovering (F1_extinct = 73.3 vs. F1_recovered = 91.6). By contrast, the SVC model achieved better identification of extinction outcomes (recall_extinct = 73.3%; F1_extinct = 75.5) with only a small trade‐off for the recovered class (F1_recovered = 91.1). Given that false negatives for extinction are especially undesirable in a risk‐prediction setting, we therefore used the SVC model for subsequent feature‐importance analyses, to prioritise predictors that are informative for extinction discrimination rather than majority‐class recovery.

### 3.4 Demography Is Best Extinction Risk Predictor, but Genomics Data Further Refines It

Extinction risk in the simulated bottleneck scenarios was strongly structured by the demographic inputs governing immediate population viability, particularly bottleneck population size (*N*_b_) and fecundity. Across the parameter grid, extinctions were concentrated in the lowest *N*_b_ and fecundity combinations, whereas populations with larger *N*_b_ and/or higher fecundity generally recovered (Figure 5a).

**FIGURE 5:**
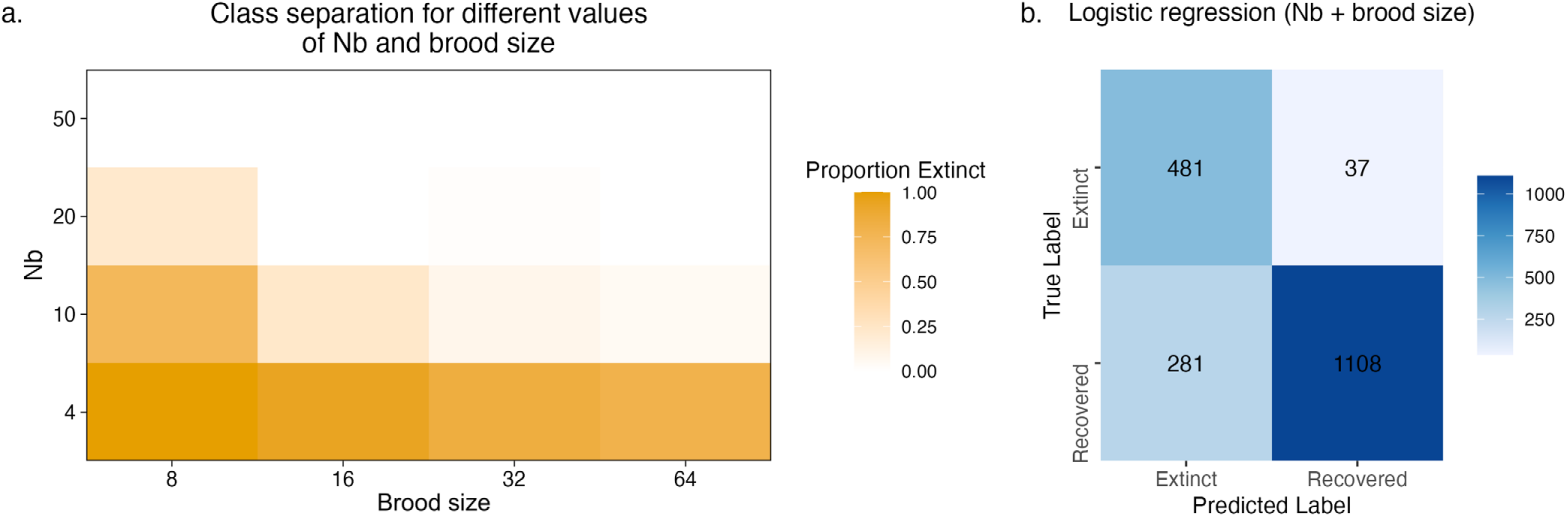
Demographic baselines for extinction‐risk prediction at the onset of the bottleneck (y0). (a) Heatmap showing the proportion of simulated populations that went extinct across combinations of bottleneck population size (*N*_b_) and fecundity. (b) Confusion matrix for a logistic regression classifier trained using only *N*_b_ and fecundity to predict extinction versus recovery on an unseen test set. Together, the panels show that *N*_b_ and fecundity explain much of the variation in extinction outcome, but that classification performance depends on class‐sensitive metrics as well as overall accuracy.

To establish a demographic baseline, we fitted a logistic regression model using only *N*_b_ and fecundity at y0. These parameters most directly determine immediate viability at bottleneck onset, whereas *N*_a_ represents historical demography and is evaluated in the broader demographic predictor analyses below (Table S2). This simple model achieved high overall accuracy on an independent test set (83.3%), but it performed poorly as a risk classifier because precision was low (i.e., many populations predicted to go extinct subsequently recovered), consistent with strong overlap among stochastic outcomes within parts of the parameter space (Figure 5b, Table 2).

We then evaluated whether including *N*_b_ and fecundity alongside genomic features improved predictive performance. Adding these demographic covariates increased overall accuracy for some algorithms, but did not consistently improve extinct‐class detection across models (Table 2). This indicates that, while *N*_b_ and fecundity capture a large fraction of the variance in extinction outcome in this simulation design, genomic summaries can provide complementary information that is not fully redundant with these demographic inputs.

Taken together, these results show that demography provides a strong extinction‐risk prediction at bottleneck onset. However, information on precision/recall/F1 needs to be accounted for to avoid over‐interpreting high accuracy driven by the recovered (majority) class. In settings where realised bottleneck severity and fecundity are uncertain or unavailable, the genomic‐only models provide an alternative route to risk discrimination. By contrast, in some ex situ settings, including those within European Association of Zoos and Aquaria (EAZA) Ex situ Programmes (EEPs), founder size may be known prospectively (van Oosterhout, Helsen, et al. 2026; van Oosterhout, Speak, et al. 2026), providing unusually detailed demographic information. Furthermore, in post‐bottleneck populations, the actual bottleneck severity can often be inferred retrospectively from historical records, pedigrees, and temporal genomic data (Groombridge et al. 2001; Jackson et al. 2022; von Seth et al. 2022; Femerling et al. 2023; Fontsere et al. 2025). This motivates the subsequent analyses of which feature‐engineered genomic summaries contribute most strongly to extinct‐class prediction.

### 3.5 Feature Importance of ROH Distribution

To interpret the supervised predictions at bottleneck onset (y0), we quantified feature importance for the best‐performing model prioritised for extinct‐class detection (SVC; Section 3.3) using permutation importance. Across repeated permutations, the SVC model relied most strongly on the high‐quantile FROH features (particularly short‐to‐intermediate ROH length cutoffs) ranking consistently among the top predictors (Figure 6a). By contrast, mean load summaries ranked substantially lower (Figure 6a), indicating that extinction discrimination depended more on within‐population ROH structure than on genome‐wide averages alone.

**FIGURE 6:**
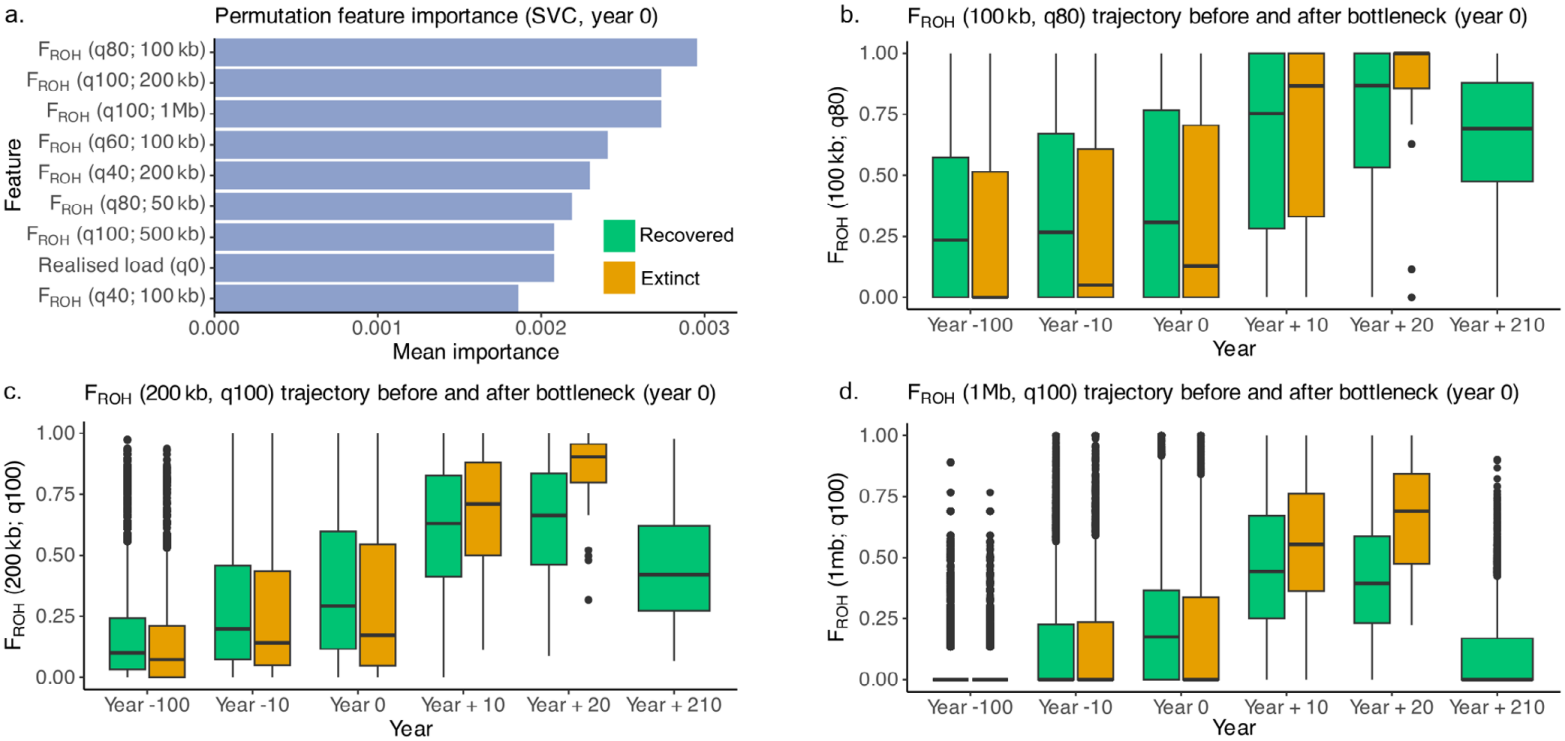
ROH‐quantile features dominate extinction‐risk prediction and show increasing outcome separation approaching bottleneck onset. (a) Permutation importance for the support vector classifier (SVC) used for extinct‐class–prioritised prediction at bottleneck onset (y0). Bars show the mean drop in model performance when each feature is randomly permuted (larger values indicate higher importance). Most top‐ranked predictors are upper‐tail summaries of ROH‐based inbreeding, FROH, expressed as quantiles across the 15 non‐overlapping 500 kb segments (e.g., FROH(q80; 100 kb) denotes the 80th percentile of segment‐level FROH computed using a minimum ROH length threshold of 100 kb; q100 corresponds to the maximum across segments). (b–d) Temporal behaviour of three top‐ranked ROH‐quantile predictors (as labelled on the *y*‐axes) across sampling timepoints relative to bottleneck onset (y‐100, y‐10, y0, y10, y20, y210). Distributions are shown separately for populations that eventually recovered (green) versus went extinct (orange). Boxplots show the median and interquartile range (IQR), whiskers extend to 1.5 × IQR, and points indicate outliers.

We next examined the temporal behaviour of the most influential ROH features across the three sampling timepoints. Trajectories showed that separation between extinct and recovered populations was modest long before the bottleneck, but became more pronounced at y0, consistent with the timing dependence observed in the benchmarking analysis (Figure 6b–d). Notably, recovered populations tended to show more consistently moderate‐to‐high values of the informative ROH quantiles at y0, whereas extinct populations exhibited greater heterogeneity, including a subset with low ROH‐quantile values despite subsequent extinction. This pattern reinforces that ROH‐based predictors summarise genomic consequences of demographic history. However, they are not deterministically linked to extinction outcome due to strong stochasticity. Together, the importance ranking and trajectory analyses identify distributional ROH summaries, rather than single mean statistics, as the most informative feature‐engineered genomic predictors for extinction‐risk classification in this simulation framework.

### 3.6 Single Reference Genomes and Minimal Data Requirements

To assess how much information is required for extinction‐risk prediction under realistic data constraints, we incorporated a complementary analysis designed to emulate the analysis of a single reference‐genome. In this framework, genomic predictors were computed from individual genomes sampled from each simulated population (i.e., ‘single‐genome’ observations) similar to the previous analyses (with the exception of population‐based metrics like nucleotide diversity). These predictors were then linked to survival/extinction outcomes, allowing us to test whether the key conclusions from population‐level summaries (Sections 3.3, 3.6) persist when genomic information is restricted to what is typically available for non‐model taxa (a reference genome plus limited individual resequencing).

We examined model performance under genomic‐only, demography‐only, and combined predictors, revealing a clear hierarchy in predictive content (Table S2). Demographic information (notably bottleneck size and related parameters) provided strong baseline discrimination (e.g., using only *N*_a_, *N*_b_, *N*_r_ and fecundity: ROC‐AUC = 0.981, PR‐AUC = 0.913; and using only population size plus *N*_a_, *N*_b_, *N*_r_ and fecundity: ROC‐AUC = 0.989, PR‐AUC = 0.947). In contrast, genomic‐only feature sets (genetic load, realised load, masked load, observed heterozygosity and FROH, without demographic covariates) performed substantially worse (ROC‐AUC = 0.769, PR‐AUC = 0.327) (Table S2). This indicates that single‐genome summaries cannot, by themselves, recover the full viability signal encoded by explicit demography in our computer simulated bottleneck scenarios. Importantly, removing bottleneck size from otherwise informative predictor sets caused a marked drop in performance (e.g., *N*_a_, *N*_r_ and fecundity only: ROC‐AUC = 0.713, PR‐AUC = 0.413; versus *N*_b_, *N*_r_ and fecundity: ROC‐AUC = 0.931, PR‐AUC = 0.775) (Table S2), underscoring that extinction in these simulations is primarily constrained by immediate demographic viability, with genomic metrics providing complementary information.

Nevertheless, the single‐genome analysis also clarifies where reference genomes add value: genomic predictors derived from single individuals retain measurable signal about historical demography and realised inbreeding (ROH/FROH), and can contribute additional discrimination when combined with even minimal demographic context. For example, adding genomic summaries to demographic predictors yielded very strong rare‐event performance (e.g., Test set 0: ROC‐AUC = 0.991, PR‐AUC = 0.955; Test set 2: ROC‐AUC = 0.990, PR‐AUC = 0.952) (Table S2), while a reduced set excluding *N*_a_, *N*_b_ and *N*_r_ but retaining population size, fecundity and genomic features performed nearly as well (Test set 8: ROC‐AUC = 0.990, PR‐AUC = 0.952) (Table S2). This suggests there is partial redundancy between demographic history parameters and information carried in population size plus genomic summaries. This supports the practical inference (see Section 1) that for data‐deficient species where rich time‐series data on demography is unavailable, single reference genomes can still inform coarse risk stratification, especially when integrated with minimal demographic context. Here, we refer to rare‐event metrics as those that emphasise performance on the minority extinct class, especially precision, recall, F1 and PR‐AUC. In other words, these metrics ask how well the model identifies the populations at risk of extinction, rather than mainly rewarding correct classification of populations that recover.

## 4 Discussion

We developed machine‐learning models that use genomic summary statistics and demographic and ecological covariates to predict extinction versus recovery. To generate training and test data with known outcomes, we used forward‐time population genomic simulations in SLiM to model replicated bottleneck scenarios and to label each simulated population as extinct or recovered. We evaluated extinction and recovery predictions across timepoints relative to a demographic bottleneck and under realistic class imbalance, drawing three main conclusions: First, the feature‐engineered genomic space is dominated by signal from historical demography, particularly ancestral population size (*N*_a_). Unsupervised learning recovers this signal but does not separate populations by eventual extinction outcome. This finding shows that the extinction signal is not linearly separable in the raw feature space, and that it is likely distributed across multiple weakly informative dimensions. Second, supervised classification becomes substantially more accurate at the onset of the bottleneck (y0), but performance is asymmetric across classes. Specifically, models readily predict recovery (the majority outcome) while extinction is harder to detect. We emphasise that this is a benchmarking result in a controlled simulated setting, rather than a claim of deterministic prediction of individual population fates in real systems. Furthermore, because both training and test data were generated within the same simulation framework, this should be interpreted as a proof of concept, rather than an external validation across species or alternative simulation models. Predicting extinction requires class‐sensitive evaluation (precision/recall/F1, and where available PR‐AUC) rather than accuracy alone. Third, demographic covariates (*N*_b_, fecundity) provide a strong baseline for predicting outcomes, while genomic summaries add complementary but limited additional signal. Feature‐importance analyses (conditioned on a classifier that better detects extinctions) indicate that distributional ROH summaries, especially upper‐quantile ROH/FROH features, carry more predictive weight than whole‐genome mean diversity or mean load metrics.

Our study corroborates the conclusions drawn by Wilder et al. (2023), who used single genomes across placental mammals and compared predictive models of conservation status based on ecological traits versus genomic features. They report that ecological data were more powerful predictors, and that models including genomic window‐based features did not outperform models with ecological variables alone. They also noted that genomic‐only models can still be informative when other data are missing. Our results show that genomic data can meaningfully complement demographic and ecological information, especially where the latter are sparse, as in many IUCN Red List assessments. The value of genomics for extinction‐risk prediction is therefore strongest when it captures information that is otherwise unavailable (e.g., recent inbreeding history). Although load‐related features were included because of their biological relevance, in this simulation framework they were less informative than ROH/FROH distributional summaries. Because deleterious mutations in the additional functional blocks contributed to fitness but were absent from the load predictors, the apparent predictive contribution of genetic load may therefore be conservative. Genomics can also serve as an initial screening tool: a standardised way to rank populations by likely risk when detailed demographic time series are missing. The output is not a final decision, but a triage step that helps prioritise which populations warrant more intensive assessment and management. This framing motivates how genome‐based predictors could be incorporated into formal assessment workflows (e.g., IUCN Red List and Green Status), which we return to below.

### 4.1 Extinction Is Harder to Predict Than Recovery

The fact that our models were relatively poor at predicting extinction is consistent with how extinction arises in small populations. Small, effectively random differences early in a bottleneck can cascade into qualitatively different population fates. Chance events are multivarious and include fewer births or a spike in deaths, biased sex ratio in the offspring, mating between closely related individuals, or the unfortunate fixation of deleterious variants (Lande 1993; Melbourne and Hastings 2008). We refer to this as an ‘extinction butterfly effect’: extinction is difficult to forecast early in the decline because stochasticity can dominate. The genomic signatures may only become apparent when the population has entered an extinction vortex. In contrast, recovery tends to be predictable when demographic conditions are permissive (e.g., larger bottleneck size and/or higher fecundity). Under this butterfly‐effect framing, extinction outcomes are intrinsically harder to classify from summary predictors because the same starting conditions can diverge rapidly due to compounded stochasticity, which both limits attainable recall for extinction and increases the importance of rare‐event evaluation metrics. Accordingly, we view the present binary classification framework as a tractable first step toward future probabilistic risk models, rather than as a final predictive framework for real populations.

In addition, the ‘route’ to extinction can be heterogeneous: multiple drivers and feedbacks can yield the same terminal outcome label, so no single predictor (genomic or demographic) will be strongly informative in every scenario (Caughley 1994; Brook et al. 2008; DiLeo et al. 2024). Although extinction is ultimately a demographic outcome, genetic factors can still contribute additional predictive information because their effects on future demographic performance may not be fully captured by the demographic covariates available at the time of prediction. The fact that predictive discrimination is strongest at bottleneck onset is encouraging, because this is often the stage at which intensive monitoring, management intervention, and DNA sampling are most likely to occur. Furthermore, the relationship between genomic summary statistics and viability is unlikely to be linear across their full range. Much of the observed variation may be effectively neutral with respect to short‐term fate, whereas small differences near critical ranges (e.g., the upper‐quantile ROH/FROH summaries) can disproportionately shift extinction risk. This helps explain why mean diversity and mean load are weak discriminators.

Within these simulated datasets, extinction was the minority class, creating a class imbalance that made extinction harder to predict. From a modelling perspective, this combination of outcome heterogeneity, weak effect sizes, and class imbalance creates two well‐known challenges for supervised learning. First, class imbalance means that many algorithms can achieve high overall accuracy by favouring the majority class (recovery) unless training, tuning and evaluation explicitly prioritise rare‐event detection (He and Garcia 2009). Second, under imbalance, metrics that more directly reflect performance on the rare class (particularly precision–recall summaries) can be more informative than ROC‐based summaries alone (Saito and Rehmsmeier 2015). These considerations also help explain why unsupervised methods do not reveal a distinct ‘extinction cluster’: extinction outcomes likely occupy overlapping regions of feature space and are characterised by weak, distributed signals across many predictors. Separation therefore emerges most clearly once outcome labels are incorporated, enabling supervised models to combine small effects across features.

### 4.2 The Importance of ROH Distributions

A consistent result across models was the importance of ROH/FROH quantile summaries, especially upper‐quantile measures, for distinguishing extinction versus recovery at bottleneck onset. ROH segments are widely used as genomic readouts of identity‐by‐descent and realised inbreeding, and their number and length distribution retain information about how recently parental lineages shared common ancestry (Kirin et al. 2010; Ceballos et al. 2018; Shafer et al. 2025). In this context, particularly q80 and sometimes q100 are informative because they reflect genomes (or genomic regions) with unusually high autozygosity, which is where recessive deleterious variants are most likely to be expressed. Indeed, long ROH have been repeatedly linked to inbreeding depression across taxa (Sams and Boyko 2019; Kyriazis et al. 2025). Furthermore, the upper‐quantile of ROH/FROH may capture one genetic component of the extinction butterfly effect, that is, rare, recent close‐kin matings that generate extreme autozygosity, which can tip small populations towards extinction.

At the same time, ROH should not be treated as a deterministic ‘red flag’. Even though ROH summaries capture an important axis of genetic risk, extinction outcomes in our simulations remain strongly shaped by demographic stochasticity (and in real populations, also by other forms of stochasticity not simulated by our model). Hence, some populations go extinct despite relatively modest ROH at y0. This supports a practical interpretation for conservation: ROH distributions are most useful as part of a multivariate risk profile (alongside bottleneck size and fecundity), and ML is valuable precisely because it can integrate these partially informative dimensions into probabilistic predictions rather than relying on single‐metric thresholds (Kyriazis et al. 2025).

### 4.3 Future Developments in AI‐Informed Conservation Genomics

The next methodological gains are likely to come less from ‘more complex models’ per se and more from how we feature engineer ML models to help them understand the genomic drivers and signatures associated with future extinction. Examining composite statistics that combine uncorrelated (or weakly correlated) dimensions of genomic and life‐history variation is particularly promising. For example, a composite metric that combines the interaction between recent inbreeding and masked load captures two dimensions that jointly determine the severity of inbreeding depression (Kyriazis et al. 2025). Recent inbreeding can be captured by ROH‐based summaries, because longer ROH reflect more recent common ancestry. In our analyses, quantile summaries were more informative than mean values, suggesting that summary statistics preserving extreme ROH/FROH values may capture recent inbreeding better than genome‐wide averages alone. Inbreeding (and drift) also interact with the masked load of partially recessive deleterious alleles at low frequency, which remains ‘hidden’ in heterozygotes until inbreeding increases homozygosity. A logical next step would be to test whether combining such ROH‐based summaries with masked‐load proxies further improves prediction. Importantly, this composite is not equivalent to either axis alone, which is exactly what ‘advanced feature engineering’ should achieve: encoding mechanistic interactions in a low‐dimensional form that generalises. Similar interactions can be expected between other metrics, such as the bottleneck size and the number of offspring per female. Severe bottlenecks (and long‐ROH) are most damaging to species with low fecundity as this reduces the population size available to purging.

Beyond the approaches benchmarked here, representation‐learning models (including neural‐network architectures) are a promising next step to evaluate for extinction‐risk prediction under rare‐event and heterogeneous failure modes. Although these models can become ‘black boxes’, recent work shows they can be paired with complementary, more transparent analyses to improve interpretability and biological insight (Winder et al. 2025).

### 4.4 Future Directions

In the next decade, the availability of reference‐quality genomes is set to expand rapidly through coordinated initiatives that aim to generate high‐quality assemblies for large fractions of eukaryotic biodiversity (e.g., the Earth BioGenome Project; Lewin et al. 2022). In parallel, formal extinction‐risk assessment remains a time‐ and expertise‐intensive process, and the Red List still covers a small and taxonomically uneven subset of described species (Rondinini et al. 2014; Bachman et al. 2019). Furthermore, Red List information is affected by knowledge gaps and taxonomic bias, reflecting large differences in research effort, monitoring and basic natural‐history knowledge among groups (Donaldson et al. 2016; Cowie et al. 2022; Borgelt et al. 2022). Genomic data can help to narrow this gap by providing standardised, comparable indicators (e.g., heterozygosity, ROH‐based inbreeding, and proxies of realised vs. masked load) that are obtainable even when demographic time series are missing. We believe this is an increasingly realistic prospect as genome resources proliferate (Shafer et al. 2015; Supple and Shapiro 2018; van Oosterhout, Helsen, et al. 2026; van Oosterhout, Speak, et al. 2026; van Oosterhout 2024; Rossetto et al. 2021). Moreover, it is timely because conservation policy and assessment frameworks are increasingly moving toward integrating genetic/genomic evidence alongside traditional criteria, and recovery‐oriented assessments (e.g., the IUCN Green Status of Species) provide a natural home for adding quantitative genomic indicators where they improve inference and decision support (Hoban et al. 2020; IUCN 2021; van Oosterhout, Helsen, et al. 2026; van Oosterhout, Speak, et al. 2026). However, our results suggest that genomic data can contribute to conservation decision support when demographic data are sparse, but they are not a replacement for formal assessment frameworks.

Our results suggest a pragmatic way forward: genomics will be most impactful where it complements sparse demography, and where ML may help integrate heterogeneous genomic summaries into calibrated probabilities that support prioritisation and triage (Wilder et al. 2023). In this study, ML combined several weak genomic signals into a single risk‐prediction framework, but the results support its use as additional decision support rather than as a replacement for Red List/Green Status criteria. This may help triage data‐deficient taxa and flag cases that warrant targeted monitoring or conservation management follow‐up. The applied value of conservation genomics would be greatly enhanced by reporting and archiving the specific metrics that are most predictive of extinction risk.

Regarding the computer simulation models, a natural next step is to extend these simulations beyond neutral and deleterious summaries by incorporating functionally and environmentally relevant variation (e.g., adaptive loci or environmental QTLs), building on recent work showing that high ancestral *N*_e_ can increase extinction risk via inbreeding depression while potentially buffering extinction risk through additive adaptive variation (Femerling et al. 2023; van Oosterhout, Helsen, et al. 2026; van Oosterhout, Speak, et al. 2026). A second priority is to explore more realistic spatial structure and metapopulation dynamics, which will be important for interpreting single‐genome predictions based on reference genomes. A third priority is to extend the selection model beyond density‐regulated hard selection by incorporating competitive filtering under soft selection, where genotypes differ in relative recruitment success rather than only absolute viability (Birley and van Oosterhout 2026). Finally, exploring a wider range of demographic and evolutionary scenarios will clarify how general these weak, overlapping genomic signals are and improve how well genome‐informed risk prediction transfers across species and contexts. ‘Digital twin’ models are particularly promising in this regard because they are designed to simulate the key demographic, ecological and genetic features of a real population. Although such digital twins cannot be developed for every population, modelling frameworks that capture the dominant drivers and predictive genomic features could still provide broadly useful decision support across many threatened taxa. In this setting, ML is well suited to integrating weak, distributed signals into standardised, updateable risk and recovery scores. In summary, considerable work remains, but we hope this paper motivates closer collaboration between conservation scientists and computational modellers to develop the next generation of genome‐informed risk assessment tools.

## 5 Conclusions

Using forward‐time simulations spanning a range of bottleneck scenarios, we show that supervised ML can predict population fate from genomic summaries. However, performance depends strongly on timing, class imbalance, and the availability of demographic context. Unsupervised structure is dominated by historical demography, whereas supervised models achieve their best discrimination at bottleneck onset, with extinction remaining intrinsically harder to detect than recovery. Across models, the most informative genomic predictors are distributional ROH/FROH features, especially upper‐quantile summaries. Taken together, our results support a pragmatic role for genomics as additional decision support: combining ROH‐based indicators and load proxies with even sparse demography may help improve triage and prioritisation, and reporting the most predictive metrics will make genome‐informed risk and recovery assessment more actionable as genomic resources expand across taxa.

## Funding

J.C.W. was supported by the UKRI Biotechnology and Biological Sciences Research Council Norwich Research Park Biosciences Doctoral Training Partnership (BB/T008717/1). H.E.M. acknowledges support from a European Research Council (ERC) Starting Grant (acronym ERODE, ‘Evolutionary dynamics of genomic erosion and its application in biodiversity conservation’), grant agreement code: 101078303. T.B. and C.v.O. acknowledge charitable funding from the Colossal Science Foundation Inc. (‘Colossal Foundation’) supporting conservation genomics research. C.v.O. also acknowledges institutional support via the Earth and Life Systems Alliance (ELSA), University of East Anglia. J.G. was supported by Research England's Expanding Excellence in England (E3) Fund, UK Research and Innovation.

## Ethics Statement

This study used in silico forward‐time population genomic simulations and associated computational analyses only; no new data were collected from humans or live animals. Accordingly, no institutional ethics approval, animal‐care protocol, or collection permits were required.

## Conflicts of Interest

The authors declare no conflicts of interest.

## Supporting information


**Figure S1:** Distributions of key genomic features in simulated flycatcher chromosomes at the start of a population bottleneck.
**Figure S2:** Genetic load expressed in lethal equivalents (LEs) before population decline across ancestral population sizes (*N*_a_) in simulated populations.
**Figure S3:** Selection coefficient (|*s*|) versus dominance coefficient (*h*) of the deleterious mutations in the SLiM simulations. Selection coefficients were drawn from a gamma distribution with mean *s* = −0.05 and shape parameter 0.5, with 5% of deleterious mutations assigned a lethal effect (*s* = −1).
**Table S1:** Hyperparameter grids used for model selection in supervised machine‐learning (ML) classifiers predicting extinction versus recovery.
**Table S2:** Predictor‐set definitions and classification performance for the single‐genome (reference‐genome–style) analysis.

## Data Availability

The Machine Learning code that supports the findings of this study is openly available in GitHub at https://github.com/jcwinder/genomic_extinctionrisk_ML/ and the SLiM code at https://osf.io/eu5dq/overview?view_only=8ae8117f194f4b8288b1bce4bddccc87.
