# Supplementary material for: Machine‐Learning Models Trained on Genomic Summary Statistics Assess Extinction Risk and Recovery Potential of Bottlenecked Populations
Source: Evol Appl. 2026 Oct 7;19(10):e70331. doi: 10.1111/eva.70331 (PMC13646493; doi:10.1111/eva.70331)
Supplement: Supplementary file 1 [file EVA-19-e70331-s001.docx]

**Supplementary Information**

**Figure S1:** Distributions of key genomic features in simulated flycatcher chromosomes at the start of a population bottleneck. Genetic load, masked load, realised load, nucleotide diversity (*π*), heterozygosity (H_o_), fraction of the genome in runs of homozygosity (F*_ROH_*) with length cutoffs of 20, 50 and 100 kb were computed for 500 kb segments of the chromosome. These segment-level summaries were used as feature-engineering inputs to test whether within-genome heterogeneity in genomic risk improved prediction beyond whole-chromosome means. F*_ROH_* with longer length cutoffs of 200 kb, 500 kb and 1 Mb were computed from the whole chromosome to capture broader-scale autozygosity across the linked scaffold.

**Supplementary Figure S2**. Genetic load expressed in lethal equivalents (LEs) before population decline across ancestral population sizes (Na) in simulated populations. Boxplots show the distribution of genetic load after burn-in for each ancestral population size (Na=100 to 10,000) reflecting mutation–selection–drift equilibrium under the simulation parameterisation. Load increases with Na, consistent with higher standing polymorphism of rare, slightly deleterious variants in larger populations (Bertorelle et al. 2022). The number of LEs is somewhat elevated in Na=100 because of the fixation of slightly deleterious mutations in the ancestral population during burn-in. Although this drift load does not contribute to inbreeding depression, it still contributes to total genetic load and may elevate extinction risk.

**Supplementary Figure S3**. Selection coefficient (|*s*|) vs dominance coefficient (*h*) of the deleterious mutations in the SLiM simulations. Selection coefficients were drawn from a gamma distribution with mean *s* = −0.05 and shape parameter 0.5, with 5% of deleterious mutations assigned a lethal effect (*s* = −1). Dominance coefficients were assigned as *h* = 0.5 exp(−13|*s*|), so that more strongly deleterious mutations were increasingly recessive (Agrawal & Whitlock 2011; Dussex et al. 2023).

**Table S1:** Hyperparameter grids used for model selection in supervised machine-learning (ML) classifiers predicting extinction versus recovery. For each method, candidate hyperparameter combinations were evaluated by grid search using 5-fold cross-validation on the training set; the best-performing configuration (as defined by the primary optimisation metric used in the analysis) was retained and evaluated on the independent test set. Not all hyperparameters apply to all methods.

| **Method** | **Parameter** | **Search space** |
| --- | --- | --- |
| **Random forest** | n_estimators | 100, 500, 1000 |
|  | max_features | None, sqrt, log2 |
| **SVC** | C | 0.1, 1, 10, 100 |
|  | gamma | 'scale', 0.01, 0.001 |
| **XGBoost** | n_estimators | 100, 200 |
|  | max_depth | 3, 5, 7 |
|  | learning_rate | 0.01, 0.1, 0.2 |
|  | subsample | 0.8, 1.0 |
| **Decision tree** | max_depth | 3, 5, 10, None |
|  | min_samples_leaf | 1, 5, 10 |
|  | max_features | None, sqrt, log2 |
| **K-nearest neighbours** | n_neighbours | 1, 3, 5, 7, 9, 11 |
|  | weights | uniform, distance |
|  | p | 1, 2 |

**Table S2:** Predictor-set definitions and classification performance for the single‑genome (reference‑genome–style) analysis. For each simulated population replicate and time point, one diploid genome was sampled and assigned the replicate’s eventual outcome (extinct vs recovered). Using an extreme gradient boosting classifier (XGBoost), we evaluated alternative predictor sets (“Test 0–8”) that partition demographic covariates, contemporary population size at the sampling time point, and genomic summary statistics (total load, realised load, masked load, observed heterozygosity and ROH‑based inbreeding, *F_ROH_*). Model selection was performed by grid search within 5‑fold cross‑validation on the training data, and performance is summarised on the independent test set using the area under the receiver operating characteristic curve (ROC‑AUC) and the area under the precision–recall curve (PR‑AUC).

| **Metric** | **Test 0** | **Test 1** | **Test 2** | **Test 3** | **Test 4** | **Test 5** | **Test 6** | **Test 7** | **Test 8** |
| --- | --- | --- | --- | --- | --- | --- | --- | --- | --- |
| **ROC-AUC** | 0.991 | 0.991 | 0.990 | 0.989 | 0.981 | 0.931 | 0.713 | 0.769 | 0.990 |
| **PR-AUC** | 0.955 | 0.953 | 0.952 | 0.947 | 0.913 | 0.775 | 0.413 | 0.327 | 0.952 |

Predictor-set definitions:

Test 0 = Population_size + genomic predictors + Na + Nb + Nr + BroodSize + Year;

Test 1 = genomic predictors + Na + Nb + Nr + BroodSize + Year;

Test 2 = Population_size + genomic predictors + Na + Nb + Nr + BroodSize (no Year);

Test 3 = Population_size + Na + Nb + Nr + BroodSize (no genomic predictors);

Test 4 = Na + Nb + Nr + BroodSize (demography only);

Test 5 = Nb + Nr + BroodSize (demography excluding Na);

Test 6 = Na + Nr + BroodSize (demography excluding Nb);

Test 7 = genomic predictors only;

Test 8 = Population_size + genomic predictors + BroodSize (excluding Na, Nb, Nr and Year).
